# BRE modulates granulosa cell death to affect ovarian follicle development and atresia in the mouse

**DOI:** 10.1038/cddis.2017.91

**Published:** 2017-03-23

**Authors:** Cheung Kwan Yeung, Guang Wang, Yao Yao, Jianxin Liang, Cheuk Yiu Tenny Chung, Manli Chuai, Kenneth Ka Ho Lee, Xuesong Yang

**Affiliations:** 1 Division of Histology and Embryology, Key Laboratory for Regenerative Medicine of the Ministry of Education, Medical College, Jinan University, Guangzhou 510632, China; 2 Chinese Medicine College, Jinan University, Guangzhou 510632, China; 3 Key Laboratory for Regenerative Medicine of the Ministry of Education, School of Biomedical Sciences, Chinese University of Hong Kong, Shatin, Hong Kong; 4 Division of Cell and Developmental Biology, University of Dundee, Dundee DD1 5EH, UK

## Abstract

The *BRE* (brain and reproductive expression) gene, highly expressed in nervous and reproductive system organs, plays an important role in modulating DNA damage repair under stress response and pathological conditions. Folliculogenesis, a process that ovarian follicle develops into maturation, is closely associated with the interaction between somatic granulosa cell and oocyte. However, the regulatory role of BRE in follicular development remains undetermined. In this context, we found that BRE is normally expressed in the oocytes and granulosa cells from the primordial follicle stage. There was a reduction in follicles number of BRE mutant (BRE^−/−^) mice. It was attributed to increase the follicular atresia in ovaries, as a result of retarded follicular development. We established that cell proliferation was inhibited, while apoptosis was markedly increased in the granulosa cells in the absence of BRE. In addition, expressions of *γ*-H2AX (marker for showing DNA double-strand breaks) and DNA damage-relevant genes are both upregulated in BRE^−/−^ mice. In sum, these results suggest that the absence of BRE, deficiency in DNA damage repair, causes increased apoptosis in granulosa cells, which in turn induces follicular atresia in BRE^−/−^ mice.

BRE is expressed in a various tissues that include the adrenal glands, brain, heart, kidneys, testis and ovaries.^1, 2^ The highest level of expression is in the nervous and reproductive systems, hence the gene was named *BRE* (brain and reproductive expressed). BRE is now considered to be an adapter protein or a homeostatic protein, which plays a role in stress response and DNA damage repair by some yet unknown mechanisms.^3^ It has been reported that homeostasis is archived by modulating the action of hormones and cytokines in physiological and various pathological conditions (including inflammation, infection and cancers).^4^ In addition, BRE also acts as the TNFRSF1A modulator, as it can modulate TNF signaling by directly binding to TNFR-1.^5^ We have recently reported that BRE plays a vital role in controlling stem cell differentiation by maintaining stemness and also neurogenesis and somitogenesis during embryo gastrulation.^6, 7^ However, little is known of the physiological and pathological function of BRE in the reproductive system, in which normally express high levels of BRE. We could cause the lack of obvious reproductive defects in BRE knockout mice for the delay of investigating BRE functions on reproductive biology. Apparently, more elaborative studies on reproductive system are indispensable to elucidate whether or not *BRE* gene have functions in reproductive activities. Hence, we generated BRE mutant mice and carefully examined the development of ovarian follicles in these mice to elucidate how the absence of BRE affects crucial events during folliculogenesis.

Ova are the female reproductive cells that reside and develop within the ovaries, a pair of ductless female reproductive glands. The ovarian follicle, as the functional unit of the ovary, is morphologically composed of an oocyte surrounded by granulosa and theca cells. These cells protect and support the development of the oocytes. Given the appropriate hormonal environment, primordial follicles are induced to develop through the primary, secondary and mature follicular stages. However, most of follicles normally degenerate to be atretic follicles, which could occur in every stages of follicular development.^8^ At birth, the ovary contains approximately one million hibernating primordial follicles and some of them become activated to undergo folliculogenesis during puberty. The various developmental stages that the activated primordial follicles pass through during folliculogenesis are also shared by many animals.

Ovarian follicle development is precisely regulated by a sequence of autocrine and paracrine factors. In addition, with input from endocrine hormones that includes pituitary and ovarian hormones. It is especially the balance of these hormones that determines whether a developing follicle becomes maturated or undergoes atresia.^9, 10, 11^ Among these hormones, follicle-stimulating hormone (FSH) is the most important, playing a role in the survival of early antral-staged follicles and the growth, activation and differentiation of prenatal follicles.^12, 13^

The cellular and molecular mechanisms that determine the developmental fate of ovarian follicles is still poorly understood.^14^ There is now accumulating evidences that indicate the death of follicular granulosa cells is partly responsible for causing follicular atresia.^15, 16^ Granulosa cells could become apoptotic by interfering with steroidogenesis and the addition of dexamethasone, which in turn trigger follicular atresia. In contrast, insulin-like growth factor (IGF) could protect the granulosa cells from apoptosis, induced by dexamethasone, which in turn represses follicular atresia.^17^ In this context, we have investigated whether BRE is involved in regulating follicular development and atresia—through its effect on granulosa cell survival. We systematically examined the development of the ovarian follicles in BRE mutant mice and specially focused on the correlation between follicular atresia and granulosa cell growth and death.

## Results

### BRE mutation reduces ovarian size and weight

**Figure 1:**
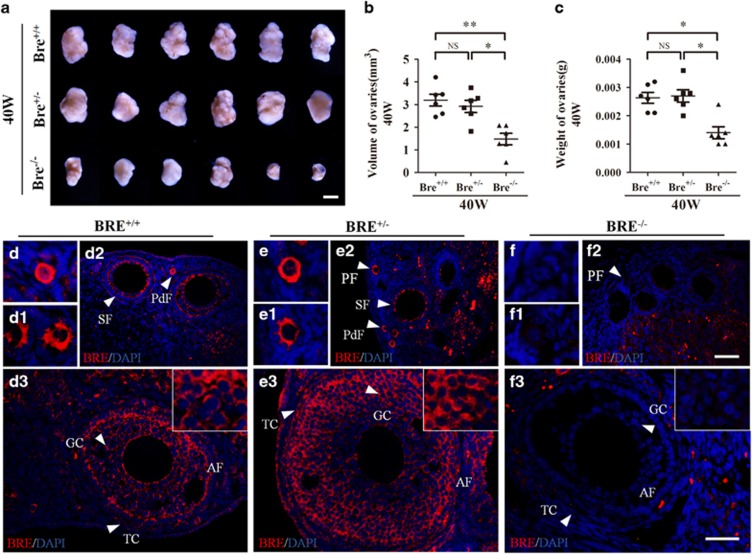
BRE knockout mice produce smaller ovaries. (**a**) Representative appearance of 40-week-old BRE^+/+^, BRE^+/−^ and BRE^−/−^ ovaries. (**b** and **c**) Plot charts comparing the ovarian volumes (**b**) and weights amongst the three groups of ovaries. (**d–f**) Representative immunofluorescent micrographs of BRE^+/+^ (**d**), BRE^+/−^ (**e**) and BRE^−/−^ (**f**) ovarian sections stained with BRE antibodies. The staining confirms that BRE^−/−^ mutant primordial (**f**), primary (**f**1), secondary (**f**2) and antral (**f**3) follicles do not express BRE. Furthermore, in BRE^+/+^ and BRE^+/−^ follicles, BRE is manly expressed in the granulosa cells (**d** and **e**). PdF, primordial follicle; PF, primary follicle; SF, secondary follicle; TC, theca cells; GC, granulosa cells. Scale bars=1000 *μ*m in **a**; 50 *μ*m in **d**2, **e**2 and **f**2; 50 *μ*m in **d**3, **e**3, **f**3

To investigate the role of BRE in ovarian development, we first measured the weights of freshly collected BRE^+/+^, BRE^+/−^ and BRE^−/−^ ovaries. Six ovaries from each group were measured and weighed (Figure 1). We determined that the average size and weight of BRE^−/−^ ovaries were both markedly reduced compared with BRE^+/−^ and BRE^+/+^ ovaries. There was no significant difference between BRE^+/−^ and BRE^+/+^ ovaries. The average volume of BRE^+/+^, BRE^+/−^ and BRE^−/−^ ovaries were 3.19±0.26, 2.91±0.27 and 1.48±0.25 mm^3^, respectively (Figure 1b). The average weight of BRE^+/+^, BRE^+/−^ and BRE^−/−^ ovaries was 2.63±0.19, 2.7±0.22 and 1.4±0.21 mg, respectively (Figure 1c).

We next examined the BRE express pattern in the ovaries using immunofluorescent staining. The result revealed that BRE was expressed in the oocytes of primordial and primary BRE^+/+^ follicles (Figures 1d and d1), and the granulosa cells of secondary and antral follicles (Figures 1d2 and d3). The expression pattern in BRE^+/−^ follicles was similar to BRE^+/+^ follicles (Figures 1e and e1). Not unexpectedly, BRE was not expressed in BRE^−/−^ ovaries, apart from autofluorescence from red blood cells (Figure 1f). The BRE expression pattern suggests that the gene might play a role in ovarian follicle development.

### BRE mutation reduces number of follicles in the ovaries

**Figure 2:**
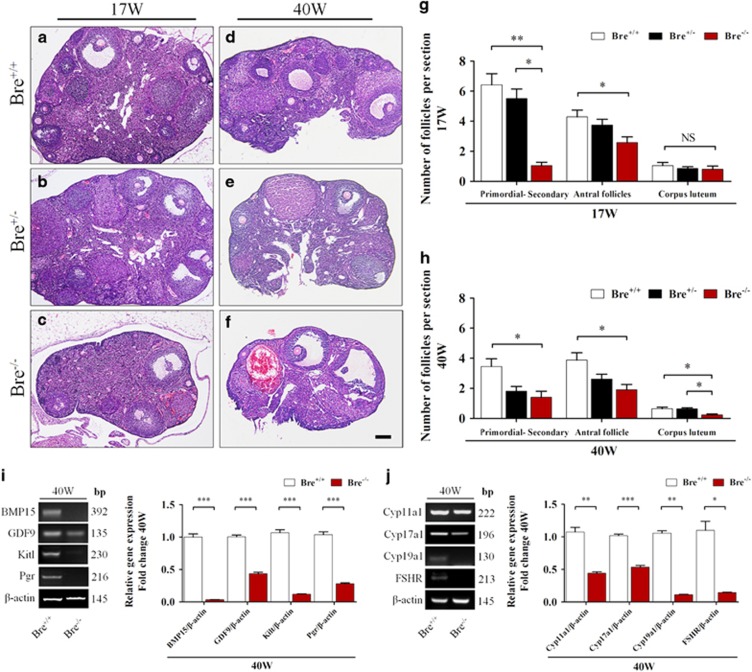
Follicle development in BRE-wild-type and -knockout ovaries. (**a–f**) Representative histological appearance of 17- and 40-week-old BRE^+/+^ (**a** and **d**), BRE^+/−^ (**b** and **e**) and BRE^−/−^ (**c** and **f**) ovaries stained with hematoxylin and eosin. (**g** and **h**) Bar charts comparing the number of follicles at different stages of development in 17- (**g**) and 40- (**h**) week-old BRE^+/+^, BRE^+/−^ and BRE^−/−^ ovaries. (**i**) Semiquantitative RT-PCR analysis revealed that *BMP15*, *GDP9*, *Kit1* and *Pgr* expression was significantly reduced in 40-week BRE^−/−^ mouse ovaries. (**j**) Semiquantitative RT-PCR analysis also showed that Cyp11a1, Cyp17a1, Cyp19a1 and FSHR expression significantly decreased. Scale bar=200 *μ*m in **a**–**f**

The numbers and types of growing follicles were estimated from serial stained sections of 17- and 40-week-old BRE^+/+^, BRE^+/−^ and BRE^−/−^ ovaries (Figures 2a–f). The results revealed that the numbers of primordial, primary, secondary and antral follicles were significantly decreased in 17-week BRE^+/−^ and BRE^−/−^ ovaries as compared with BRE^+/+^ ovaries. However, there was no significant difference in the number of corpus luteum found between the three groups. It was estimated that in 17-week-old ovaries, the average number of follicles were significantly reduced in BRE^−/−^ mice compared with BRE^+/+^ mice (*P*<0.05; for primordial–secondary follicles: in BRE^+/+^=6.4±0.7, BRE^+/−^=5.5±0.6 and BRE^−/−^=1.0±0.2; for antral follicles: BRE^+/+^=4.3±0.4, BRE^+/−^=3.7±0.4 and BRE^−/−^=2.6±0.4; for corpus luteum: BRE^+/+^=1.0±0.2, BRE^+/−^=0.9±0.1 and BRE^−/−^=0.8±0.2, *n*=6 in each group, Figure 2g). The types of follicles found in 40-week-old ovaries were also counted. It was determined that the numbers of follicles and corpus luteum were significantly reduced in BRE^−/−^ ovaries as compared with BRE^+/+^ (for primordial–secondary follicles: BRE^+/+^=3.5±0.5, BRE^+/−^=1.8±0.3 and BRE^−/−^=1.4±0.4. For antral follicles: BRE^+/+^=3.9±0.5, BRE^+/−^=2.6±0.3 and BRE^−/−^=1.9±0.3. For corpus luteum: BRE^+/+^=0.6±0.1, BRE^+/−^=0.6±0.1 and BRE^−/−^=0.2±0.1, *n*=6, *P*<0.05, Figure 2h).

Using RT-PCR approach, we examined the expressions of genes known to be associated with follicular development, in 40-week-old BRE^+/+^ and BRE^−/−^ ovaries. The results demonstrated that bone morphogenetic protein 15 (BMP15), growth differentiation factor 9 (GDF9), Kit1, Pgr, Cyp11a1, Cyp17a1, Cyp19a1 and FSHR expressions were significantly repressed in BRE^−/−^ ovaries compared to their wild-type counterpart (BMP15, ***P<0.001; GDF9, ***P<0.001; Kit1, ***P<0.001; Pgr, ***P<0.001; Cyp11a1, **P<0.01; Cyp17a1, ***P<0.001; Cyp19a1, **P<0.01; FSHR, *P<0.05; *n*=3 for each group, Figures 2i and j). The results suggest that BRE is required for follicle development and maturation, and the blocking of these processes would lead to follicle atresia.

### BRE mutation enhances follicular atresia

**Figure 3:**
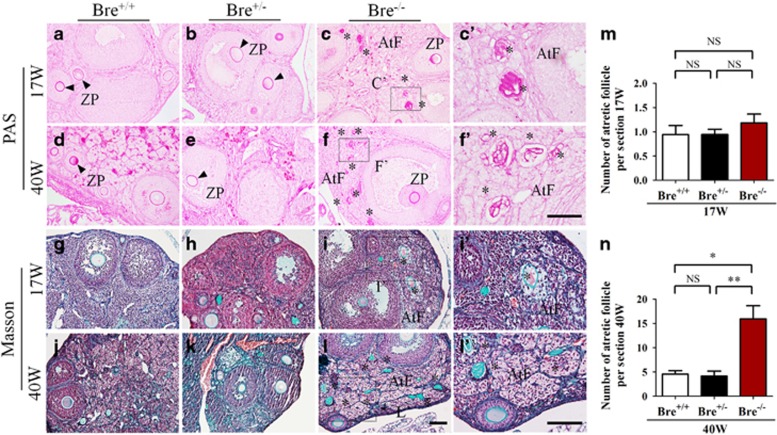
Ovarian sections stained with specialized dyes. Representative micrographs of 17- and 40-week-old ovarian sections stained with PAS (**a–f**′) and Masson's trichrome stain (**g–l**′). (**i**′ and **l**′) Higher-magnification images of the regions highlighted by the dotted squares in **i** and **l**, respectively. (**m** and **n**) Bar charts comparing the number of atretic follicles in 17- (**m**) and 40- (**n**) week-old BRE^+/+^, BRE^+/−^ and BRE^−/−^ ovaries. AtF, atretic follicle; ZP, zona pellucida. Scale bar=100 *μ*m in **a**–**l**; 50 *μ*m in **c**′ and **f**′ 100 *μ*m in **i**′ and **l**′

Histological sections of 17 (young)- and 40 (old)-week-old BRE^+/+^, BRE^+/−^ and BRE^−/−^ ovaries were stained with Masson and periodic acid Schiff (PAS) staining to reveal the extent of atresia in these ovaries (Figure 3). The high magnification of the zona pellucida shows its distinct contraction in the atretic follicles, as demonstrated in Figures 3c′. Using these as markers, we methodically counted the numbers of atretic follicles present in the three groups of ovaries. The results showed that the number of atretic follicles increased in young BRE^−/−^ ovaries (17 weeks old: BRE^+/+^=0.9±0.2, BRE^+/−^=0.9±0.1 and BRE^−/−^=1.2±0.2 *n*=6, *P*>0.05, Figure 3m). The number of atretic follicles were significantly increased in old BRE^−/−^ ovaries (40 weeks old: BRE^+/+^=4.6±0.7, BRE^+/−^=4.2±1.0 and BRE^−/−^=15.9±2.7, *n*=6, **P<0.01, *P<0.05; Figure 3n).

### BRE mutation represses granulosa cell proliferation

**Figure 4:**
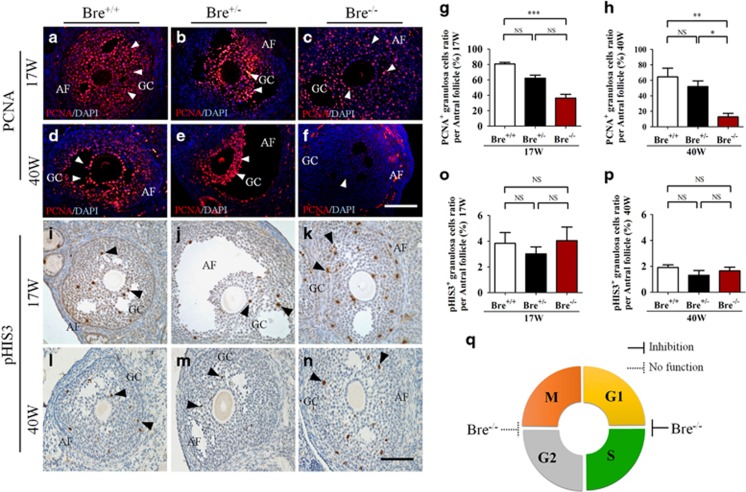
Ovarian sections immunofluorescently stained for PCNA and pHIS3. Representative micrographs of developing follicles stained for PCNA (**a–f**) and pHIS3 (**i–n**) to demonstrate the extent of granulosa cell proliferation in the follicles. (**g** and **h**) Bar charts comparing the average number of PCNA^+^ granulosa cells in antral follicles of 17- (**g**) and 40- (**h**) week-old BRE^+/+^, BRE^+/−^ and BRE^−/−^ ovaries. (**o** and **p**) Bar charts comparing the average number of pHIS3^+^ granulosa cells in antral follicles. (**q**) Pie chart predicting where BRE targets the cell cycle during granulosa cell proliferation (as predicted from the BRE^−/−^ results). AF, antral follicle; GC, granulosa cells. Scale bar=100 *μ*m in **a**–**n**

The relationship between BRE and granulosa cell proliferation was investigated. Longitudinal sections of 17- and 40-week-old BRE^+/+^, BRE^+/−^ and BRE^−/−^ ovaries were immunofluorescently stained with proliferating cell nuclear antigen (PCNA) antibody (Figures 4a–f). Examination revealed that the number of PCNA^+^ granulosa cells (arrows) in both 17- and 40-week BRE^−/−^ ovaries were significantly reduced compared with BRE^+/+^ ovaries (PCNA, 17 weeks: BRE^+/+^=80.75±2.16, BRE^+/−^=62.13±4.07 and BRE^−/−^=36.44±4.95, *n*=6, ***P<0.001, Figure 4g; 40 weeks: BRE^+/+^=64.53±11.18, BRE^+/−^=51.94±7.26 and BRE^−/−^=12.77±4.49, *n*=6, **P<0.01, *P<0.05, Figure 4h). In addition, the number of BrdU^+^ granulosa cells were significantly decreased compared with 17-week-old BRE^+/+^ ovaries (BrdU, BRE^+/+^=52.26±2.16, BRE^−/−^=16.46±1.90, *n*=6, ***P<0.001, Supplementary Figures S3A–D). The results indicated that the number of replicating granulosa cells was significantly reduced in the absence of BRE (Figure 4q). Using the same strategy, we also immunohistochemical stained the sections using pHIS3 antibody (Figures 4i–n). Interestingly, we did not find that any significant difference between the number of pHIS3^+^ granulosa cells (arrows) in both 17- and 40-week-old BRE^−/−^ and BRE^+/+^ mouse ovaries (pHIS3, 17 weeks: BRE^+/+^=3.84±0.84, BRE^+/−^=3.02±0.54 and BRE^−/−^=4.04±1.06, *n*=6, Figure 4o; 40 weeks: BRE^+/+^=1.92±0.19, BRE^+/−^=1.31±0.37 and BRE^−/−^=1.66±0.28, *P*>0.05, Figure 4p). This might imply that BRE may mainly exerting its effect at the G1–S phase of the cell cycle (Figure 4q).

In order to determine how BRE targets the cell cycle during granulosa cell proliferation, we silenced the BRE expression levels through transfection with Control-siRNA or BRE-siRNA in COV434 cells. We can found more necrotic cells in the BRE-siRNA compared with the Control-siRNA group (black arrows, Supplementary Figures S3E and F). Quantitative PCR (qPCR) analysis was also performed to confirm that the expression level of BRE was downregulated after BRE-siRNA transfection 48 h (Supplementary Figure S3G). To test whether the cell cycle progression pattern accounts for the marked differences after BRE-siRNA trasfection, we analyzed cell cycle patterns by propidium iodide (PI) staining. After BRE-siRNA transfection 48 h, we found COV434 cells showed a unique cell cycle pattern with a high percentage of G1 cells (Control-siRNA=58.73±0.89, BRE-siRNA=72.50±4.12, *n*=3, *P<0.05, Supplementary Figure S3H) and low percentage of G2/M cells (Control-siRNA=8.72±1.64, BRE-siRNA=0.49±0.49, *n*=3, **P<0.01, Supplementary Figure S3H). After BRE-siRNA transfection 72 h, we found COV434 cells showed a unique cell cycle pattern with a high percentage of G1 cells (Control-siRNA=59.37±0.23, BRE-siRNA=64.03±0.67, *n*=3, **P<0.01, Supplementary Figure S3I), low percentage of S cells (Control-siRNA=37.93±0.95, BRE-siRNA=24.57±0.77, *n*=3, ***P<0.001, Supplementary Figure S3I) and high percentage of G2/M cells (Control-siRNA=2.67±0.80, BRE-siRNA=11.40±1.42, *n*=3, **P<0.01, Supplementary Figure S3I). We also detected the expression levels of cell cycle-related genes using qPCR. The results showed that downregulating RBE with BRE-siRNA transfection significantly changed the expression levels of CyclinA, CyclinB, CyclinD1 and p53 compared with the Control-siRNA group. However, the CyclinE1 was significantly downregulated by BRE-siRNA transfection (CyclinA, *P*>0.05; CyclinB, *P*>0.05; CyclinD1, *P*>0.05; CyclinE1, *P<0.05; *n*=9 for each group, Supplementary Figure S3J). Although upregulating genes included p21 and p27 (p21, ***P<0.001; p27, *P<0.05; *n*=9 for each group, Supplementary Figure S3J), indicating that the change in the cell cycle-related gene expression might partially be responsible for the phenotype induced by silencing BRE.

### BRE mutation enhances granulosa cell death

**Figure 5:**
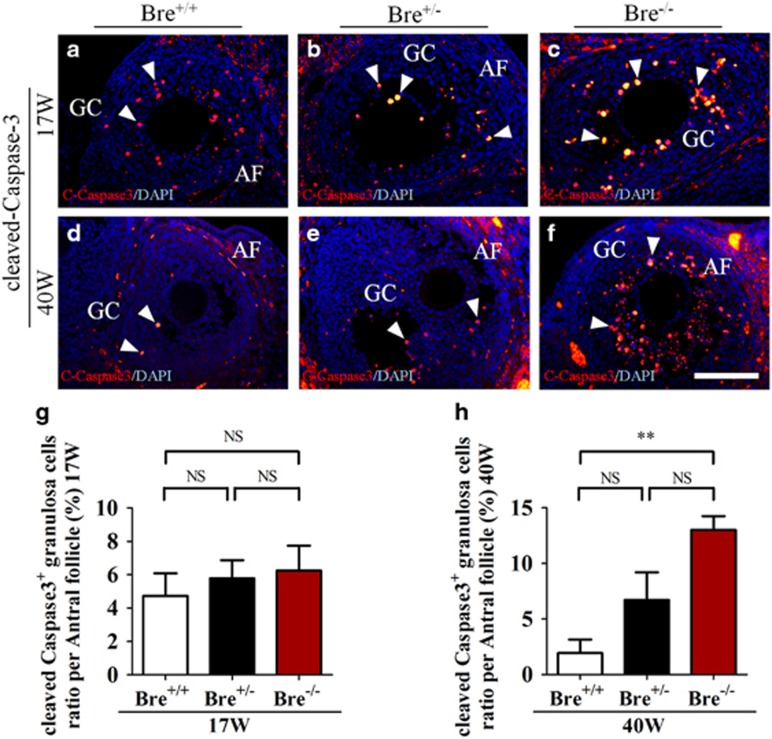
Ovarian sections stained for cleaved-Capase-3. (**a–f**) Representative micrographs of antral follicles immunofluorescently stained for c-Capase3 in 17- (**a**–**c**) and 40- (**d**–**f**) week-old BRE^+/+^, BRE^+/−^ and BRE^−/−^ ovaries. (**g** and **h**) Bar charts showing the average number of c-Capase3^+^ granulosa numbers in antral follicles from 17- (**g**) and 40- (**h**) week-old BRE^+/+^, BRE^+/−^ and BRE^−/−^ ovaries. AF, antral follicle; GC, granulosa cells. Scale bar=100 *μ*m in **a**–**f**

We investigated whether BRE was involved in regulating apoptosis in the follicular granulosa cells. This was archived by immunofluorescently staining sections, prepared from 17- and 40-week-old (BRE^+/+^, BRE^+/−^ and BRE^−/−^) ovaries, with cleaved Caspase-3 (C-Caspase3) antibodies (Figures 5a–f). The result showed that the number of C-Caspase3^+^ granulosa cells (indicated by arrows) were significantly increased compared with 40-week-old BRE^+/+^ ovaries (C-Caspase3 17 weeks: BRE^+/+^=4.73±1.35, BRE^+/−^=5.78±1.10 and BRE^−/−^=6.25±1.48, *n*=6, Figure 5g; 40 weeks: BRE^+/+^=1.93±1.23, BRE^+/−^=6.72±2.50 and BRE^−/−^=13.04±1.23, *n*=6, **P<0.01, Figure 5h). The results indicate that there were significantly more apoptotic granulosa cells in the BRE mutants in 40 weeks.

**Figure 6:**
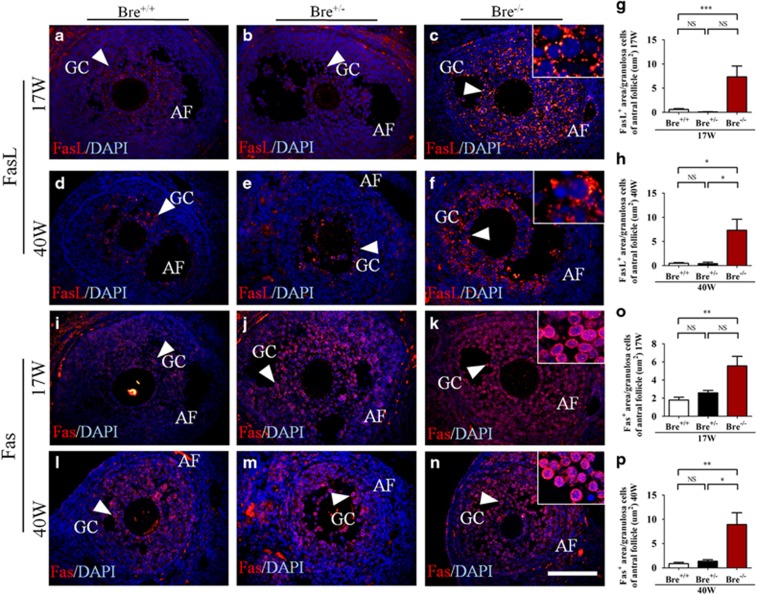
Ovarian sections stained for FasL and Fas expression. Representative micrographs of antral follicles immunofluorescently stained for FasL (**a–f**) and Fas (**i–n**) expression in 17- and 40-week-old BRE^+/+^, BRE^+/−^ and BRE^−/−^ ovaries. Higher-magnification images are highlighted at top right corners of **c**, **f**, **k** and **n**. (**g** and **h**) Bar charts showing areas that are FasL^+^ over the total granulosa cell area of antral follicles, in 17- (G) and or 40- (**h**) week-old BRE^+/+^, BRE^+/−^ and BRE^−/−^ ovaries. (**o** and **p**) Bar charts showing areas that are Fas^+^ over the total granulosa cell area of antral follicles, in 17- (**o**) and or 40- (**p**) week-old BRE^+/+^, BRE^+/−^ and BRE^−/−^ ovaries. AF, antral follicle; GC, granulosa cells. Scale bar=100 *μ*m in **a**–**n**

Seventeen- and forty-week-old ovarian sections produced from BRE^+/+^, BRE^+/−^ and BRE^−/−^ mice were also immunofluorescently stained for FasL (Figures 6a–f) and Fas (Figures 6i–n). These two proteins play important roles in regulating apoptosis. The stained sections were analyzed to determine the ratio of FasL^+^ and Fas^+^ area (arrows) over the total area of granulosa cells. Statistical analysis revealed that the ratios for both FasL^+^ and Fas^+^ were significantly increased in comparison with BRE^+/+^ mice (FasL^+^ 17 weeks: BRE^+/+^=0.60±0.16, BRE^+/−^=0.10±0.03 and BRE^−/−^=7.34±2.26, *n*=6, ***P<0.001, Figure 6g; FasL^+^ 40 weeks: BRE^+/+^=0.48±0.16, BRE^+/−^=0.41±0.30 and BRE^−/−^=7.34±2.26, *n*=6, *P<0.05, Figure 6h; Fas^+^ 17 weeks: BRE^+/+^=1.79±0.31, BRE^+/−^=2.59±0.27 and BRE^−/−^=5.55±1.06, *n*=6, **P<0.01, Figure 6o; Fas^+^ 40 weeks: BRE^+/+^=0.86±0.27, BRE^+/−^=1.36±0.33 and BRE^−/−^=8.94±2.44, *n*=6, **P<0.01, *P<0.05, Figure 6p). The results suggest that the Fas/FasL signaling pathway is involved in the regulation of cell apoptosis modulated by BRE.

### BRE mutation increases DNA damage susceptibility in granulosa cells

**Figure 7:**
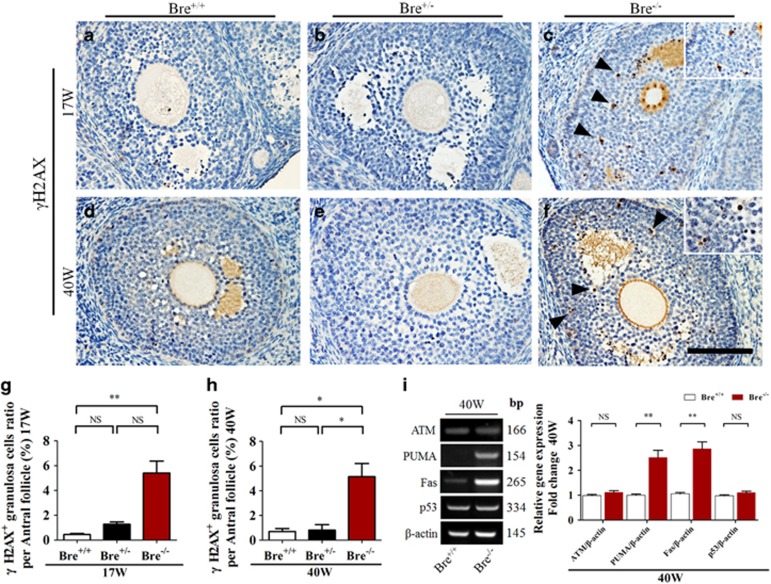
Ovarian sections stained for *γ*-H2AX. (**a–f**) Representative micrographs of 17- and 40-week-old BRE^+/+^, BRE^+/−^ and BRE^−/−^ ovarian sections immunohistochemically stained for *γ*-H2AX (marker for DNA double-strand breaks). Higher-magnification image at the top right corner is taken from **c** and **f**. (**g** and **h**) Bar charts comparing the average number of *γ*-H2AX^+^granulosa cells in antral follicles of 17- (**g**) and 40- (**h**) week-old BRE^+/+^, BRE^+/−^ and BRE^−/−^ ovaries. (**i**) Semiquantitative RT-PCR analysis showing ATM, PUMA, Fas and p53 expression in 40-week-old BRE^+/+^ and BRE^−/−^ ovaries. The accompanying bar chart indicates the normalized and averaged expression of these genes. Scale bar=100 *μ*m in **a**–**f**

Seventeen- and forty-week-old BRE^+/+^, BRE^+/−^ and BRE^−/−^ ovaries were immunohistochemically stained using *γ*-H2AX to establish whether BRE was involved in DNA damage repair (Figures 7a–f). The results clearly demonstrated that the number of *γ*-H2AX^+^granulosa cells (indicated by arrows) in both 17- and 40-week-old BRE^−/−^ ovaries was significantly increased in comparison with control BRE^+/+^ ovaries (*γ*-H2AX 17 weeks BRE^+/+^=0.45±0.08, BRE^+/−^=1.28±0.19 and BRE^−/−^=5.40±0.97, *n*=6, **P<0.01, Figure 7g; 40 weeks: BRE^+/+^=0.69±0.24, BRE^+/−^=0.81±0.45 and BRE^−/−^=5.10±1.07, *n*=6, **P<0.05, Figure 7h). Expression of genes associated with DNA damage repair (ATM, PUMA, Fas and p53) was also examined by semiquantitative RT-PCR. The results showed that PUMA and Fas expression markedly increased in the absence of BRE, whereas there was no significant change in ATM and p53 expression (ATM, *P*>0.05; PUMA, **P<0.01; Fas, **P<0.01; p53, *P*>0.05; *n*=3 for each group, Figure 7i). These findings suggest that, in the absence of BRE, there was significantly more DNA damage in the granulosa cells.

## Discussion

In this study, we first examined and compared the morphology of wide-type and BRE knockout ovaries—as BRE is highly expressed in the reproductive system. We determined that the average weight and size of the BRE mutant ovaries were significantly smaller than wild-type ovaries—even though there was no difference between the overall weights of these mice. This suggests that BRE might play a specific role in ovarian follicle development. BRE immunofluorescent staining revealed that BRE was strongly expressed in the oocyte at primordial follicle stage and then it was reverted to the granulosa cells that surround the oocytes at later stages. The granulosa cells of BRE mutants did not express BRE and suggests that it maybe the potential cause of the smaller ovarian size and weight. Moreover, we carefully assessed the distribution of follicles, at various stages of development, in BRE^+/+^, BRE^+/−^ and BRE^−/−^ ovaries, and determined that there were significantly fewer follicles (at all developmental stages) in the BRE mutant ovaries. Hence, we investigated whether the reduction in the number of follicles was attributed to reduced cell proliferation, enhanced apoptosis or a combination of both.

Folliculogenesis involves the activation of a small number of primordial follicles, which then develop and pass through the primary, secondary, antral and follicle stages. Only a few of these mature follicles are ovulated, whereas the majority normally undergoes atresia in the mouse. For follicles to develop normally to maturity and not undergo atresia, it involves very precise cellular and molecular interactions.^18, 19^ It has been reported that BMP15 and GDF9 were important for inducing follicular cells to differentiate during follicle development and maturity. Our RT-PCR analysis revealed that both Bmp15 and GDF9 expression was significantly reduced in BRE mutant ovarian tissues. Furthermore, Kitl, Pgr and FGFR (follicles development-relevant genes) expression was also repressed.^20^ We propose that the abnormal expression pattern of these genes maybe one of the reason why there were fewer developing follicles in the BRE mutant. However, it is generally recognized that the interaction of autocrine and paracrine effectors, including FSH and LH, ultimately determines the developmental fate of the developing follicles.^18, 19^

Our PAS and Masson's trichrome staining revealed that there were more atretic follicles in the BRE mutants, which may explain why there were fewer follicles in these mutant ovaries. We tried to establish why there were more atretic follicles by focusing on the granulosa cells. These cells are normally indispensable for inducing and supporting the development of the follicles.^21, 22^ In this context, we first examine how the absence of BRE affected granulosa cell proliferation. Immunofluorescent staining was performed on an ovarian section using PCNA and pHIS3 antibodies. We found that there were significantly fewer PCNA^+^ granulosa cells in 17- and 40-week-old BRE^−/−^ follicles than BRE^+/+^ follicles. We also found that BrdU^+^ granulosa cells were markedly decreased in 17-week-old BRE^−/−^ antral follicles compared with the BRE^+/+^ group. Nevertheless, there was no significant difference in the numbers of pHIS3^+^ cells between the two groups. Using flow cytometry of PI staining, the cell cycle profile of COV434 cells following BRE-siRNA transfection was revealed, which confirmed that the proportion of cells during S phase was reduced after 72-h transfection. The discordance between PCNA, BrdU, pHIS3 expressions and cell cycle analysis suggested that BRE mainly plays a role in the G1–S phase of the cell cycle, which was confirmed by the experimental results of Kim *et al.*^21^ Cyclins are a family of proteins that control the cell progression through the cell cycle.^22^ Transitions between the different phases of the cell cycle are governed by positive (cyclins and cyclin-dependent kinases (CDK)) and negative (CDK inhibitors) cell cycle regulatory proteins.^23^ CyclinE1, a functional complex as a subunit of CDK2, is required for the G1/S cell cycle transition. Our experiments suggest that silencing BRE can activate the expression of CDK inhibitors p21 and p27, which can block the cell cycle through inhibiting of CyclinE1.

Undoubtedly, the extent of granulosa cell proliferation and apoptosis affects the number of viable follicles that develop to the antral stage.^21^ Increased granulosa cell death is most likely to be the cellular mechanism that directly or indirectly induces follicle atresia.^24^ Quirk *et al.* have reported that IGF-I and estradiol could promote bovine granulosa cell proliferation and survival because of their increased resistance to apoptosis.^25^ It has been demonstrated that cell cycle arrest and excessive apoptosis of granulosa cells during follicle development could be induced by a high-fat diet. Similarly, we have demonstrated that granulosa cell death (indicated by c-Capase3 labeling) significantly increased in 40-week-old ovaries in the absence of BRE. This implies that BRE conferred an increase in resistance to apoptosis in granulosa cells under normal physiological conditions. Moreover, we have found high level of Fas and FasL expression in BRE^−/−^ mouse ovaries – indicating that Fas-FasL signaling has been activated to induce apoptosis in the granulosa cells. The significance of Fas-FasL signaling in granulosa cells has already been confirmed.

Next, we asked the question why granulosa cell death was increased in the absence of BRE expression. It has been reported that BRE is mainly involved in DNA damage repair and stress response. Therefore, we decided to determine the presence of *γ*-H2AX foci, a marker of DNA double-strand breaks,^26^ in BRE^−/−^ and BRE^+/+^ ovaries. We established that there were significantly more *γ*-H2AX cells in BRE^−/−^ than in BRE^+/+^ follicles. Moreover, we also revealed that ATM, PUMA, Fas and p53 (DNA damage repair-relevant genes)^27^ expression was significantly upregulated in BRE^−/−^ ovarian tissues. These findings implied that increased granulosa cell death in BRE mutant follicles maybe attributed to an excessive accumulation of DNA damage—as BRE is not there to assist in DNA repair.

**Figure 8:**
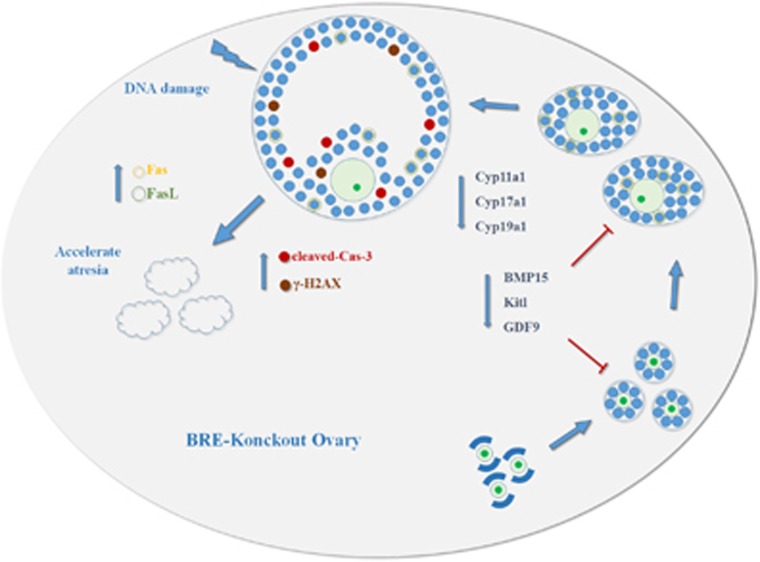
Model depicting how BRE might play a role in the regulation of mouse folliculogenesis and follicular atresia

We have schematically illustrated in Figure 8 how we believe BRE influences follicle development and atresia. Briefly, the illustration shows that BRE is mainly expressed in oocyte of primordial follicles, and then it is the specifically expressed granulosa cells that surround the primary, secondary and antral follicles. The expression pattern indicates that BRE might exert its role in folliculogenesis and follicular atresia via the granulosa cells. Moreover, when granulosa cells cannot express BRE, it would lead to growth arrest at G1 and G2/M phase, and enhances excess apoptosis (as cells are less efficient in conducting DNA repair). These events in turn induce the follicles to undergo atresia. Nevertheless, there are still more experiments that need to be conducted before we can completely understand how BRE functions in the female reproductive biology.

## Materials and methods

### Mice

BRE-wild-type (Bre^+/+^), -heterozygote (Bre^+/−^) and -knockout (Bre^−/−^) mice were obtained from the Chinese University of Hong Kong Animal Centre and maintained at 25 °C on a 12 h light/dark cycle. Bre^−/−^ mutant mice were generated based on the Cre/LoxP recombination against a C57/BL/6J background. The BRE targeted strain (B6Dnk; B6N-Bre^tm1a (EUCOMM)Wtsi/H^), in which the exon 3 of *BRE* gene is flanked by two loxP sites, was purchased from European Conditional Mouse Mutagenesis Programme (EUCOMM). TNAP-Cre mice (129-Alpl^tm1 (cre) Nagy^/J, stock number: 008569, Jackson Laboratory, Bar Harbor, ME, USA), which are primordial germ cell-specific transgenic mice, were used to cross with female BRE^fx/fx^ mice to generate BRE^−/−^ mice. All of the mice were maintained under a 12 light/12 dark cycle at a constant temperature of ~23 °C and humidity between 35 and 75%. All animal procedures were approved by AEEC (Animal Experimentation Ethics Committee) of Chinese University of Hong Kong and Hong Kong Government Department of Health. The animal experiments were conducted in accordance with the approved guidelines.^28^

### Cell culture and gene transfection

COV434 (human ovarian granulosa cells) was attained from GuangZhou Jennio Biotech Co., Ltd, China (Guangzhou, Guangdong, China). The cells were cultured in a humidified incubator with 5% CO_2_ at 37 °C in six-well plates (1 × 10^5^ cells per ml) containing HAM'S/F-12 (Myclone, Logan, UT, USA) supplemented with 10% fetal bovine serum (Gibco, Gaithersburg, MD, USA). For the gene transfection, the COV434 cells were transfected by Control-siRNA (5′-AAGCCUCGAAAUAUCUCCU-3′) or BRE-siRNA (5′-CTGGACTGGTGAATTTTCA-3′), with the help of lipofectamin 3000 (Invitrogen, Carlsbad, CA, USA).^4^ Cells were plated to 50–70% confluence at the time of transfection and the preparation of siRNA–lipid complexes, which were subsequently added to the cells.

### Cell cycle analysis

COV343 cells were transfected with Control-siRNA and BRE-siRNA. At 48 and 72 h after transfection, the cells were trypsinized, fixed in 70% ethanol and stored at 4 °C for overnight. After washing with PBS, the cells were stained with PI solution (0.04 mg/ml of PI, 0.25 mg/ml of RNase A, 0.1% Triton X in PBS) for 15 min, and then subjected to flow cytometry analysis (Beckman Coulter Gallios flow cytometer, Pasadena, CA, USA). DNA content was determined on counting 10 000 cells. Percentage of cells in each cell cycle phase was analyzed using MultiCycle for Windows software (Redmond, WA, USA).

### Histology

Briefly, 17- and 40-week-old Bre^+/+^, Bre^+/−^ and Bre^−/−^ ovaries were fixed in 4% paraformaldehyde at 4 °C for 24 h. The specimens were then dehydrated, cleared in xylene and embedded in paraffin wax. The embedded specimens were serially sectioned at 5 *μ*m using a rotary microtome (Leica, Frankfurt, Germany). The sections were either stained with hematoxylin and eosin, PAS reaction or Masson's trichrome dyes.^29^ The sections were also immunohistochemically stained. The PAS and Masson staining were used to reveal the presence of atretic follicle in the ovarian sections. The stained histological sections were photographed using an epifluorescence microscope and an attached camera (Olympus IX51, Tokyo, Japan; Leica DM 4000B, Frankfurt, Germany) at × 200 magnification.

### Classification of developing follicles in ovarian sections

The follicles in the ovarian histological sections were developmentally staged according to their morphology as: primordial, primary, secondary, antral or atretic follicles. Briefly, an oocyte surrounded by a single layer of squamous granulosa cells was classified as a primordial follicle. Oocyte surrounded by a single or several layer/s of cuboidal granulosa cells was classified as a primary or secondary follicle, respectively. When an antrum is present, it was described as an antral follicle. The presence of zona pellucida remnants was classified as an end-stage atretic follicle.^30^ Every fifth and sixth histological sections were selected for comparison and evaluation. Follicles were only counted if they appeared in one histological section but not in the other.^30^

### Immunohistological staining

Sections of mouse ovaries were dewaxed, hydrated, incubated in citrate buffer (pH 6.0) and then heated in a microwave for antigen retrieval. Immunofluorescent staining was conducted on these treated sections using various antibodies. Briefly, the sections were incubated in the following primary antibodies diluted using PBT-NGS: BRE (1:100, Cell Signaling Technology, Boston, MA, USA), (PCNA; 1:400, Santa Cruz Biotechnology, Santa Cruz, CA, USA), p-Histone H3 (pHIS3; 1:400, Santa Cruz Biotechnology), C-Caspase3 (1:100, Cell Signaling Technology), *γ*-H2AX (1:100, Millipore, Billerica, Massachusetts, USA), Fas (1:100, Boster, Wuhan, Hubei, China), FasL (1:100, Boster) CD34 (1:100, Abcam, Cambridge, UK) or *α*-SMA (1:100 Abcam) at 4 °C overnight. Following three 5 min washes in PBS, the sections were further incubated with goat anti-rabbit IgG conjugated Alexa Fluor 555 (1:1000, Life Technologies, Carlsbad, CA, USA) for 1 h to detect the presence of BRE, PCNA, C-Caspase3, Fas or FasL. The sections were counterstained with DAPI (1:1000, Life Technologies) at room temperature for 30 min before examination. For immunohistochemical staining, secondary HRP-linked goat anti-rabbit IgG (1:300, Cell Signaling Technology) was used to reveal pHIS3, CD34 and *α*-SMA staining, or secondary HRP-linked goat anti-mouse IgG (1:300, Cell Signaling Technology) to show *γ*-H2AX staining. Daminobenzidine tetrahydrochloride substrate (DAB kit, MXB, Fuzhou, China) was used to visualize the immunostaining. Photographs were taken of the stained histological sections using an epifluorescence microscope (Olympus IX51, Leica DM 4000B) at × 200 magnification.

### *In vivo* BrdU labeling

BRE-wild-type (Bre^+/+^) and -knockout (Bre^−/−^) mice were injected with BrdU (100 mg/kg of body weight) at 10 mg/ml in PBS intraperitoneally for 2 h before killing. The ovaries were then extracted and fixed in 4% paraformaldehyde at 4 °C for 16 h. For histology, the fixed ovaries were washed, dehydrated and embedded in paraffin wax. The paraffin-embedded ovaries were serially sectioned at 4 *μ*m. The sections were immunohistologically stained using rat anti-BrdU antibody (1:200; Abcam) and counterstained with hematoxylin for morphological observation. The extent of follicular cell proliferation within follicles was determined by the presence of BrdU^+^ in randomly selected ovarian sections.

### RNA isolation and PCR analysis

Total RNA was isolated from 17- and 40-week-old Bre^+/+^, Bre^+/−^ and Bre^−/−^ ovaries or COV434 cells using Trizol (Invitrogen) according to the manufacturer's instructions. Three ovaries from each group were used. First-strand cDNA was synthesized at a final volume of 25 *μ*l using a SuperScript III First-Strand kit (Invitrogen). Following reverse transcription, RT-PCR amplification of the cDNA was performed using specific primers (Supplementary Figure 1). PCR was performed in a Bio-Rad S1000 Thermal cycler (Bio-Rad, Richmond, CA, USA). The cDNAs were amplified for 30 cycles. One round of amplification was performed at 98 °C for 10 s, at 60 °C for 15 s and then at 72 °C for 30 s (Takara, Tokyo, Japan). The PCR products (20 μl) were resolved on a 2% agarose gels (Biowest, Madrid, Spain) in 1 × TAE buffer (0.04 M trisacetate and 0.001 M EDTA) plus GeneGreen Nucleic Acid Dye (TIANGEN, Beijing, China). The reaction products were visualized using a transilluminator (Syngene, Cambridge, UK) and a computer-assisted gel documentation system (Syngene). qPCR analysis was also performed by SYBR Premix Ex Tag (Takara) using a 7900HT Fast Real-Time PCR system (Applied Biosystems, Foster City, CA, USA). The primer sets used for the qPCR are provided in Supplementary Figure 2. Each of these experiments was replicated at least three times. The RT-qPCR results were produced from four independent sets of experiments. The housekeeping gene, *β-actin*, was run in parallel to confirm that equal amounts of RNA were used in each reaction. The ratio intensity for the fluorescently stained bands of genes of interest, and *β*-actin was calculated and normalized to quantify the level of gene expression.

### Image acquisition and analysis

Whole ovaries were photographed using a fluorescence stereomicroscope (Olympus MVX10) and analyzed imaging software (Image-Pro Plus 6.0 Media Cybernetics, Media Cybernetics, MD, USA). Sections of the stained ovaries were photographed using an epi-fluorescent microscope (Olympus IX51, Leica DM 4000B) at × 200 and × 400 magnification, and analyzed using an Olympus software (Leica CW4000 FISH).

For quantification of proliferation, apoptosis and DNA damage, we counted the number of PCNA^+^, pHIS3^+^, C-Caspase3^+^ and *γ*-H2AX^+^ granulosa cells versus total DAPI^+^ granulosa cells for each antral follicle in 17- and 40-week-old mouse ovaries (Bre^+/+^, Bre^+/−^ and Bre^−/−^). The results were then compared between each group with the follicles only at the same developmental stage. For immunofluorescent staining of 17–40-week-old ovaries, total Fas^+^, FasL^+^, CD34^+^ and *α*-SMA^+^ granulosa or theca cells in antral follicles were counted.^31^ Statistical analysis was performed using a SPSS 19.0 software (SPSS software, Armonk, NY, USA), and the data were presented as mean±S.E.M. Six ovaries of each group (Bre^+/+^, Bre^+/−^ and Bre^−/−^) were used.

### Data analysis

Data analyses and construction of statistical charts were performed using GraphPad Prism 5 software (GraphPad Software, La Jolla, CA, USA). The results were presented as the mean value (x̄±S.E.M.). Statistical analysis was performed using IBM SPSS Statistics 19.0 software (IBM SPSS Statistics software, Armonk, NY, USA). Statistical significance was determined using an independent sample's *t*-test, and non-parametric independent samples Kruskal–Wallis test. *P*<0.05 was considered to be statistically significant.
