# Supplementary figures and images for: BRE modulates granulosa cell death to affect ovarian follicle development and atresia in the mouse
Source: Cell Death Dis. 2017 Mar 23;8(3):e2697–. doi: 10.1038/cddis.2017.91 (PMC5386581; doi:10.1038/cddis.2017.91)

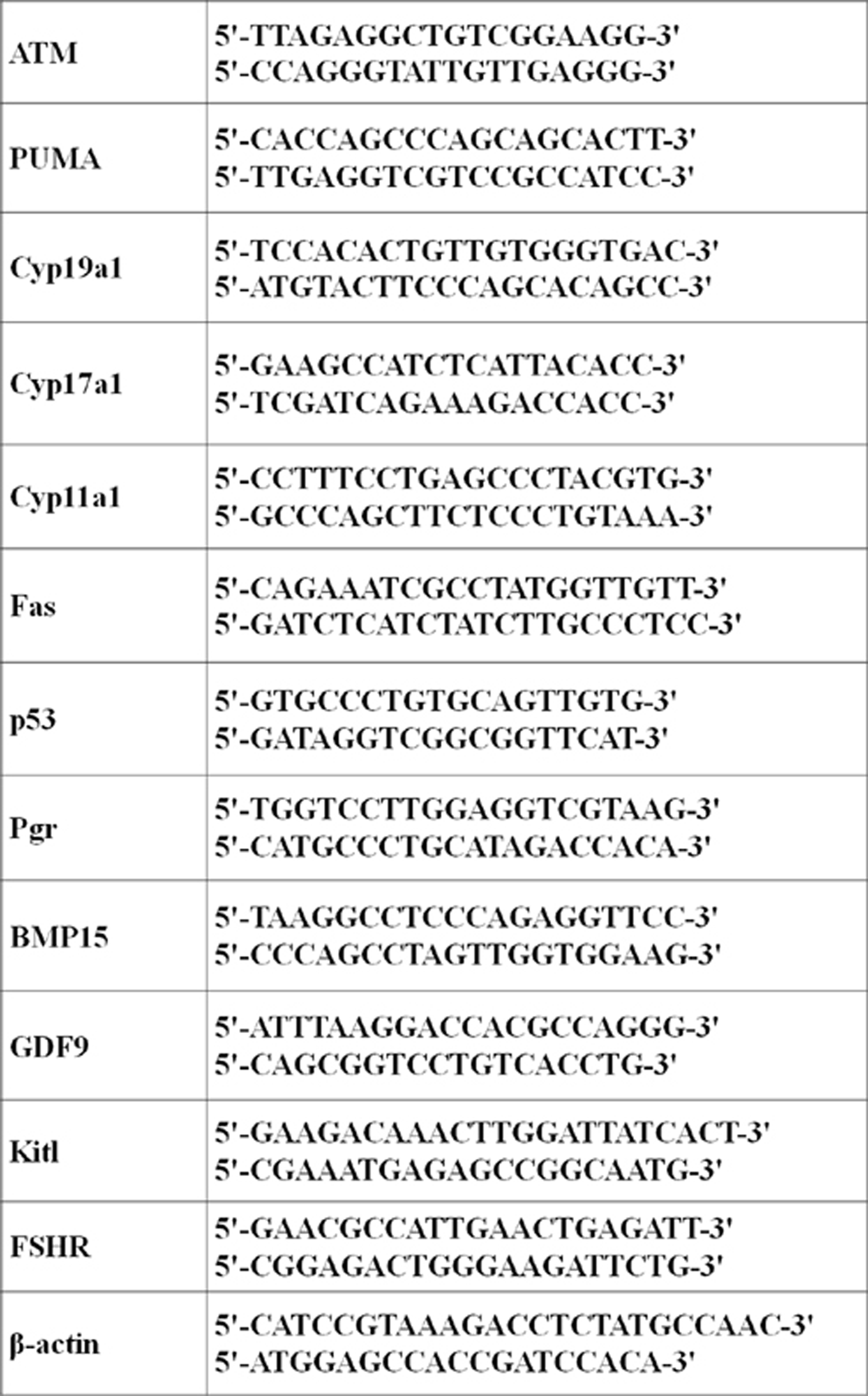

Supplement: Supplementary Figure 1 [file cddis201791x1.tif]

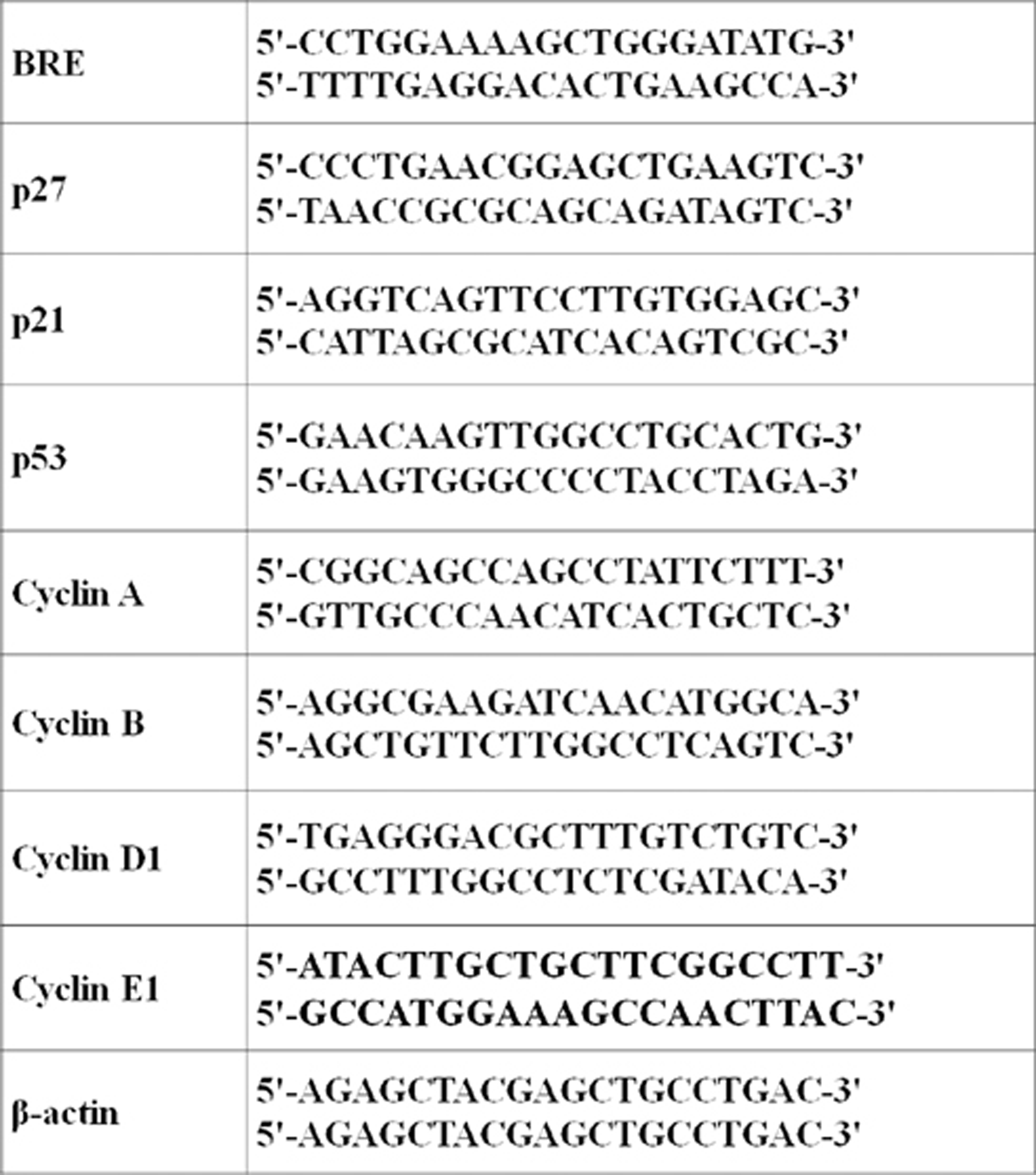

Supplement: Supplementary Figure 2 [file cddis201791x2.tif]
